# Sniff adjustment in an odor discrimination task in the rat: analytical or synthetic strategy?

**DOI:** 10.3389/fnbeh.2014.00145

**Published:** 2014-05-05

**Authors:** Emmanuelle Courtiol, Laura Lefèvre, Samuel Garcia, Marc Thévenet, Belkacem Messaoudi, Nathalie Buonviso

**Affiliations:** Centre de Recherche en Neurosciences de Lyon, Equipe Olfaction: du codage à la mémoire, CNRS UMR 5292—INSERM U1028—Université Lyon1Lyon, France

**Keywords:** sniffing, olfaction, rat, sorption properties, discrimination, olfactomotor act

## Abstract

A growing body of evidence suggests that sniffing is not only the mode of delivery for odorant molecules but also contributes to olfactory perception. However, the precise role of sniffing variations remains unknown. The zonation hypothesis suggests that animals use sniffing variations to optimize the deposition of odorant molecules on the most receptive areas of the olfactory epithelium (OE). Sniffing would thus depend on the physicochemical properties of odorants, particularly their sorption. Rojas-Líbano and Kay (2012) tested this hypothesis and showed that rats used different sniff strategies when they had to target a high-sorption (HS) molecule or a low-sorption (LS) molecule in a binary mixture. Which sniffing strategy is used by rats when they are confronted to discrimination between two similarly sorbent odorants remains unanswered. Particularly, is sniffing adjusted independently for each odorant according to its sorption properties (analytical processing), or is sniffing adjusted based on the pairing context (synthetic processing)? We tested these hypotheses on rats performing a two-alternative choice discrimination of odorants with similar sorption properties. We recorded sniffing in a non-invasive manner using whole-body plethysmography during the behavioral task. We found that sniffing variations were not only a matter of odorant sorption properties and that the same odorant was sniffed differently depending on the odor pair in which it was presented. These results suggest that rather than being adjusted analytically, sniffing is instead adjusted synthetically and depends on the pair of odorants presented during the discrimination task. Our results show that sniffing is a specific sensorimotor act that depends on complex synthetic processes.

## Introduction

Sampling of sensory information is achieved through dedicated motor systems. In olfaction, sniffing allows a rhythmic sampling of the environment and constrains both the timing and the intensity of the input to the olfactory structures. A remarkable feature of sniffing is its highly dynamic nature; sniffing in rats varies both in frequency and flow rate (Youngentob et al., 1987). This characteristic raises the important question of what the implications of these variations are on olfactory processing. The zonation hypothesis, proposed by Schoenfeld and Cleland (2005, 2006), is based on the observation that the olfactory epithelium (OE) is activated by both imposed and inherent patterns (for review, see Scott, 2006). Imposed patterns are determined by a complex interplay between the sorption properties of odorant molecules and the rate of nasal airflow, which affects the deposition of molecules across the OE (Mozell, 1964a,b; Mozell and Jagodowicz, 1973). In contrast, inherent patterns are determined by populations of olfactory neurons with different receptive properties distributed in distinct regions of the OE (Moulton, 1976; Kent and Mozell, 1992; Vassar et al., 1993; Freitag et al., 1995; Yoshihara and Mori, 1997). Hence, modifications of sniffing parameters affect the deposition of odorant molecules and the activation of the OE (Ezeh et al., 1995; Scott-Johnson et al., 2000; Scott et al., 2006; Yang et al., 2007; Scott et al., 2014). This body of evidence led Schoenfeld and Cleland (2005, 2006) to propose that during odor discrimination, an animal may adapt its sniffing parameters to optimize the deposition of odorant molecules on the most receptive OE areas. Sniffing would thus depend on the physicochemical properties of the odorant molecules, particularly their sorption (which depends notably on their water solubility and volatility). Rojas-Líbano and Kay (2012) tested this hypothesis and showed that rats used different sniff strategies when targeting a high-sorption (HS) molecule or a low-sorption (LS) molecule in a binary mixture. However, the strategy used for sniff adjustment when animal is confronted to a choice discrimination between two similarly sorbent odorants is still unknown. Indeed, at least two alternative strategies might exist for performing a two-alternative choice odor discrimination: (1) sniffing is adjusted independently for each odorant according to its sorption properties (analytical processing), or (2) sniffing is not adjusted independently for each odorant but is instead adjusted based on the pairing context (synthetic processing). The goal of this report was to test these possible sniffing strategies in the rat. We developed a method to non-invasively record sniffing to maintain physiological sniffing dynamics (Teichner, 1966). We used whole-body plethysmography with a two-alternative choice odor discrimination (Uchida and Mainen, 2003). We showed that similar sorption properties did not inevitably endow molecules with the property to be similarly sniffed. Moreover, we showed that the same odorant was sniffed differently depending on the odor pair in which it was presented. These results suggest that sniffing is adjusted in a synthetic manner that is dependent on the context in which the odorant is presented.

## Materials and methods

### Animals

Data were obtained from five male Long–Evans rats (Charles River, l'Arbresle, France) that weighed 250–300 g at the start of experimentation. Animals were housed individually at 23°C and were maintained under a 12 h light–dark cycle (lights on from 6:00 a.m. to 6:00 p.m.). Food was available *ad libitum* during the experiment. Rats were placed under water restriction, with access to water provided during the behavioral session and for 1 h after each session. Experiments were performed in strict accordance with the European Community Council directive of November 24, 1986 (86/609/EEC), and the guidelines of the French Ethical Committee and French Legislation.

### Sniff recording

**Figure 1:**
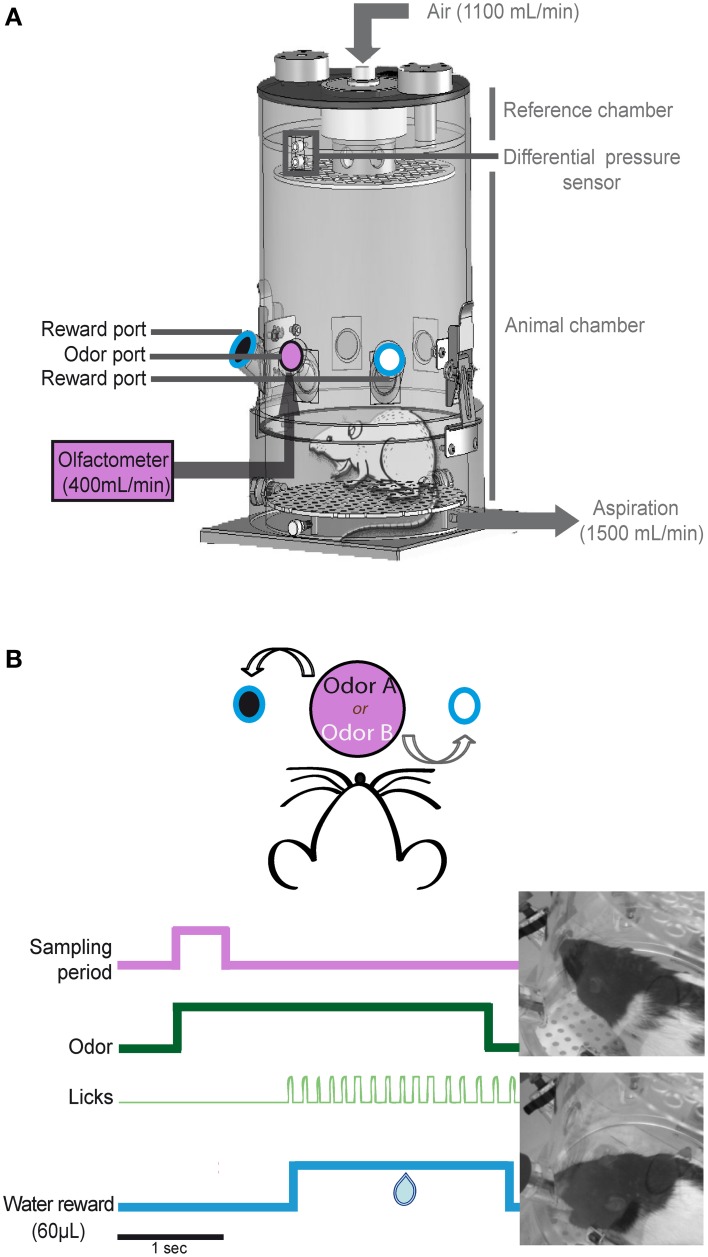
**Non-invasive sniff recording. (A)** Schematic representation of the whole-body plethysmography. This setup allows us to access the respiratory signal of the animal in a non-invasive manner. The plethysmograph is equipped with three ports: an odor port (pink, central) connected to the olfactometer and two reward ports (black and white circles surrounded with blue lines) equidistant from the central odor port. **(B)** Example of one trial. The rat starts the trial by poking its nose into the central port; this motion triggers the delivery of an odorant for 3 s (green). Each odorant was associated with a reward port: odorant A/left port and odorant B/right port. If the rat makes the correct choice, water is available at the reward port for 6 s from the beginning of the trial. The first lick (light green) triggers the delivery of 60 μL of water (blue).

The recording apparatus consisted of a whole-body customized plethysmograph (diameter: 20 cm, height: 30 cm, EMKA Technologies, France; Figure 1A) placed in a homemade sound-attenuating cage (length: 60 cm, width: 60 cm, height: 70 cm). The apparatus was composed of two independent airtight chambers: the animal chamber and the reference chamber. The pressure changes that resulted from animal respiration were measured by a differential pressure transducer (Model dpt, EMKA Technologies) with one sensor in the animal chamber and another in the reference chamber. The measured signal was amplified, digitally sampled at 1 kHz and acquired with a PC using an acquisition card (MC-1608FS, Measurement Computing, USA).

The whole-body plethysmograph was equipped with three ports (inner diameter: 2 cm, depth: 2.5 cm; Figure 1A). The odor port was elevated 8 cm from the floor and was between two reward ports that were located 6 cm to the left and right of the odor port. This central odor port was connected to a homemade olfactometer. Odorants were delivered at a constant flow of 400 mL/min. Constant deodorized air also flowed through the top of the plethysmograph at a rate of 1100 mL/min.

To maintain a constant flow through the plethysmograph and to leave the sniffing signal undisturbed, a ventilation pump was connected to the whole body plethysmograph to vacuum out the equivalent of the air pushed into the chamber at 1500 mL/min (1100 + 400 mL/min).

The reward ports contained a pipette connected to a water pump. Each port was equipped with a capacitive sensor that allowed nose poke detection in the odor port and lick detection in each of the reward ports.

### Behavioral training

#### Task

We adapted a two-alternative choice odor discrimination task developed by Uchida and Mainen (2003). Rats started a trial by poking their nose into the central port. The nose poke triggered the delivery of an odorant for 3 s. Each odorant of an odor pair was associated with a reward port: odorant A/left port and odorant B/right port (Figure 1B). The rat had 6 s to reach a reward port and lick for water. If the rat made the correct choice, the first lick triggered the delivery of 60 μL of water for 2 s. The inter-trial interval was at least 7 s. Each day, the rats performed a session with an odor pair. Each session was composed of 60–100 trials.

Animals were considered successful at discriminating an odor pair when they achieved the training criterion of 70% correct trials for each reward port on two consecutive days.

#### Odorants

We used the following odorants at saturated vapor pressure (Sigma-Aldrich, Fluka): methyl benzoate (mbz), ethyl benzoate (etbz), both enantiomers of carvone (L-car/D-car), both enantiomers of limonene (L-lim/D-lim), isoamyl acetate (iso), heptanol (hept), cumene (cum), and cyclooctane (cyc). Odorants were classified as LS or HS, as in Rojas-Líbano and Kay (2012). The sorption coefficients for all molecules are listed in Table 1. The odor pairs used were (Table 1):

- Two pairs of enantiomers: LS/LS molecules L-lim/D-lim and HS/HS molecules L-car/D-car. Enantiomers are well suited to form similar odor pairs because they possess the same physicochemical properties (Table 1) and evoke closely related responses in the OE (Schoenfeld and Cleland, 2005, 2006; Scott, 2006).
- Two pairs of non-enantiomeric odorants with similar sorption properties and comparable vapor pressures: cum/cyc (LS/LS) and hept/mbz (HS/HS).
- One pair of odorants with similar sorption properties (HS/HS) but different vapor pressures: hept/iso.
- We also tested whether an odorant induced a specific sniffing pattern or whether the pattern varied based on the pair in which the odorant was presented. For this purpose, we used the same odorant in the following pairs: D-car in the L-car/D-car pair and in the D-car/D-lim pair; hept in the hept/iso pair and in the hept/mbz pair and D-lim in the L-lim/D-lim pair and in the D-car/D-lim pair.

**Table 1:** **Physicochemical properties of the odorant molecules: vapor pressure (VP, in mm Hg at 25°C), Henry's law constant (K_aw1_ from the group method and K_aw2_ from the bond method; in atm-m^3^/mole) and S (sorptiveness)**.

**Odor**	**Carvone pair**		**Limonene pair**	
	**L-car**	**D-car**	**L-lim**	**D-lim**
VP	0.13	0.13	1.45	1.45
K_AW1_	–	–	–	–
K_AW2_	7.73*10^−5^	7.73*10^−5^	3.8*10^−1^	3.8*10^−1^
S	HS	HS	LS	LS
**Odor**	**hept/iso pair**		**hept/mbz pair**	
	**hept**	**iso**	**hept**	**mbz**
VP	0.299	5.67	0.299	0.379
K_AW1_	2.74*10^−5^	5.35*10^−4^	2.74*10^−5^	1.81*10^−5^
K_AW2_	2.34*10^−5^	5.45*10^−4^	2.34*10^−5^	3.47*10^−5^
S	HS	HS	HS	HS
**Odor**	**cum/cyc pair**		**D-car/D-lim pair**	
	**cum**	**cyc**	**D-car**	**D-lim**
VP	3.59	5.37	0.13	1.45
K_AW1_	1.05*10^−2^	4.5*10^−1^	–	–
K_AW2_	1.23*10^−2^	3.88*10^−1^	7.73*10^−5^	3.8*10^−1^
S	LS	LS	HS	LS

All parameters were obtained from ChemSpider. Data were generated from the ACD/Labs PhysChem Suite and US Environmental Protection Agency's EPISuite^tm^. The odorants used are L-carvone (L-car), D-carvone (D-car), L-limonene (L-lim), D-limonene (D-lim), heptanol (hept), methyl benzoate (mbz), isoamyl acetate (iso), cumene (cum), and cyclooctane (cyc). Odorants were classified either as low sorption (LS) or high sorption (HS).

In the discrimination task, seven pairs of odorants were used. All rats first learned the rule of the task with the odor pair mbz/etbz. Data acquired with this pair were not included in the analysis. The other odor pairs were randomly presented once the rats reached the criterion performance.

### Data processing

The sampling duration (Sd), time of odorant delivery, number of licks on each reward port, time of water delivery, and respiratory signal were recorded and stored in an SQL database using OpenElectrophy (Garcia and Fourcaud-Trocmé, 2009).

#### Respiratory signal

**Figure 2:**
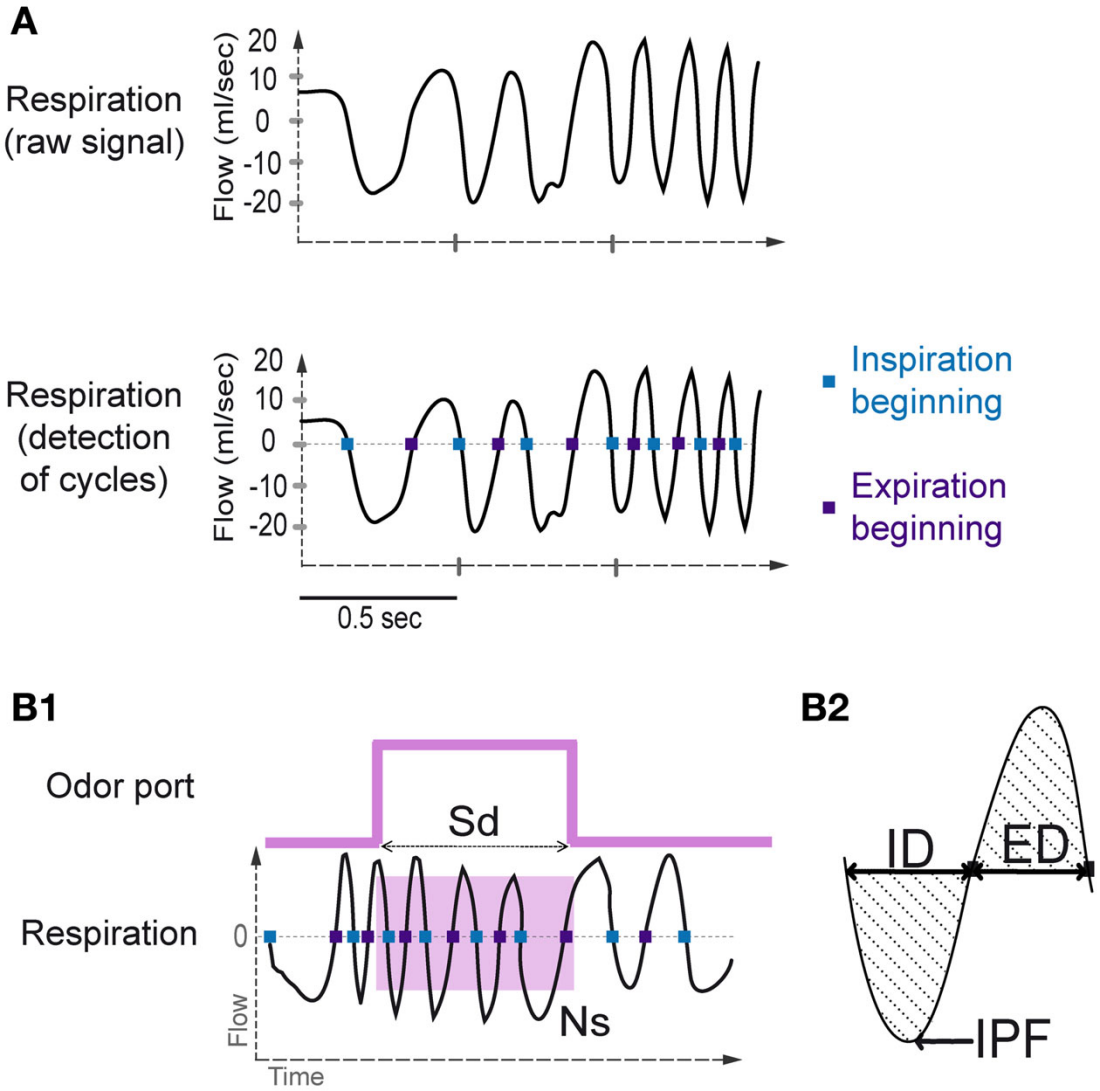
**Sniffing signal processing. (A)** Top: Raw sniffing signal recorded by the plethysmograph. An algorithm was applied to detect the zero-crossing points. Bottom: The blue squares represent the detection of the beginning of the inspiratory phase, and the violet squares represent the beginning of the expiratory phase. **(B1)** Sampling duration and number of sniffs. Sampling duration (Sd) is defined as the time spent in the odor port. The number of sniffs (Ns) is defined as the number of sniffs occurring during the sampling period (pink square). **(B2)** A representative sniff cycle is shown to illustrate the parameters measured: inspiration duration (ID), expiration duration (ED), and inspiration peak flow rate (IPF).

Using the whole-body plethysmography setup, the natural respiratory signal was a periodic function showing alternating negative (inspiration) and positive (expiration) deflections (Figure 2A). A key aspect of respiratory signal analysis was the detection of these deflections to measure respiratory cycles, which was achieved using an algorithm described in Roux et al. (2006). The algorithm performed signal smoothing for noise reduction and detection of zero-crossing points to accurately define the inspiration and expiration phases. The inspiration phase started at the zero-crossing point of the falling phase and ended at the zero-crossing point of the rising phase. The expiration phase started at the zero-crossing point of the rising phase and ended at the zero-crossing point of the falling phase (Figure 2A). In addition, to eliminate detection artifacts, we determined a cut-off value for signal duration (rejection if value < median/4) and for signal amplitude (rejection if value < median/6).

#### Sniffing parameters

Before each session, the plethysmograph was calibrated by pushing 1 mL of air into the rat chamber. The resulting pressure variation in the experimental box was recorded, allowing measurement of the respiratory volume. Respiratory cycles were measured during the sampling period (Sd, Figure 2B1), and the number of sniffs (Ns, Figure 2B1) during this period was collected. Sniffs that were considered as occurring during the sampling period were cycles in which the inspiration was included in the sampling period (Figure 2B1). For each respiratory cycle, inspiration peak flow rate (IPF), inspiration duration (ID), and expiration duration (ED; Figure 2B2) were measured. To compare the values between rats and across time, we performed data normalization for each cycle by dividing the value of each parameter for each sniff by the mean value of each parameter over the whole session.

### Statistics

Python scripts (scipy.stats) and Statview were used for all statistical analyses. We first measured the average duration of the sampling period for all the experimental sessions. We removed trials whose duration was greater than the mean sampling time plus 2 standard deviations.

The analysis focused on the respiratory signals of sessions in which the animals achieved ≥70% accuracy. Only periods resulting in a correct behavioral response were considered. To compare sniffing or sampling parameters between two odorants, we used a paired *t*-test, with the level of significance set at 0.05 (^*^p < 0.05, ^**^p < 0.01, and ^***^p < 0.001).

### Validation

To validate our setup, we presented D-lim at two different concentrations. As demonstrated previously (Youngentob et al., 1987), we found that a decrease in odorant concentration led to an increase in IPF (data not shown). This control shows that our experimental setup allowed us to accurately measure sniffing and reproduce data obtained by others.

## Results

We measured sniffing in unrestrained animals performing a two-alternative choice odor discrimination task (Figure 1). Global sniffing strategies were measured according to the Sd (time spent in the odor port) and the Ns (number of sniffs occurring during the Sd, Figure 2B1). We also individually analyzed each sniff cycle during the Sd. For all pairs combined, animals sampled odorants with 3.3 ± 0.026 sniffs (mean ± s.e.m.). We therefore focused our analysis on the first three sniffs following the odor onset (first, second, and third sniffs). For these three cycles, we analyzed the normalized ID, the normalized IPF, and the normalized ED (Figure 2B2).

Sniffing parameters were compared between odorants presented in a pair of odorants with similar sorption properties. Pairs were either HS enantiomeric (L-car/D-car), LS enantiomeric (L-lim/D-lim), HS non-enantiomeric (hept/mbz and hept/iso, with comparable and different vapor pressures in the odor pair, respectively), or LS non-enantiomeric (cum/cyc) molecules. On the whole, five pairs of odorants with similar sorption properties were tested. To test the effect of the context, we additionally used the odor pair D-car/D-lim.

### Odorant sorption and sniff adjustment: an analytical strategy is not supported

#### Pairs of enantiomeric odorants

**Figure 3:**
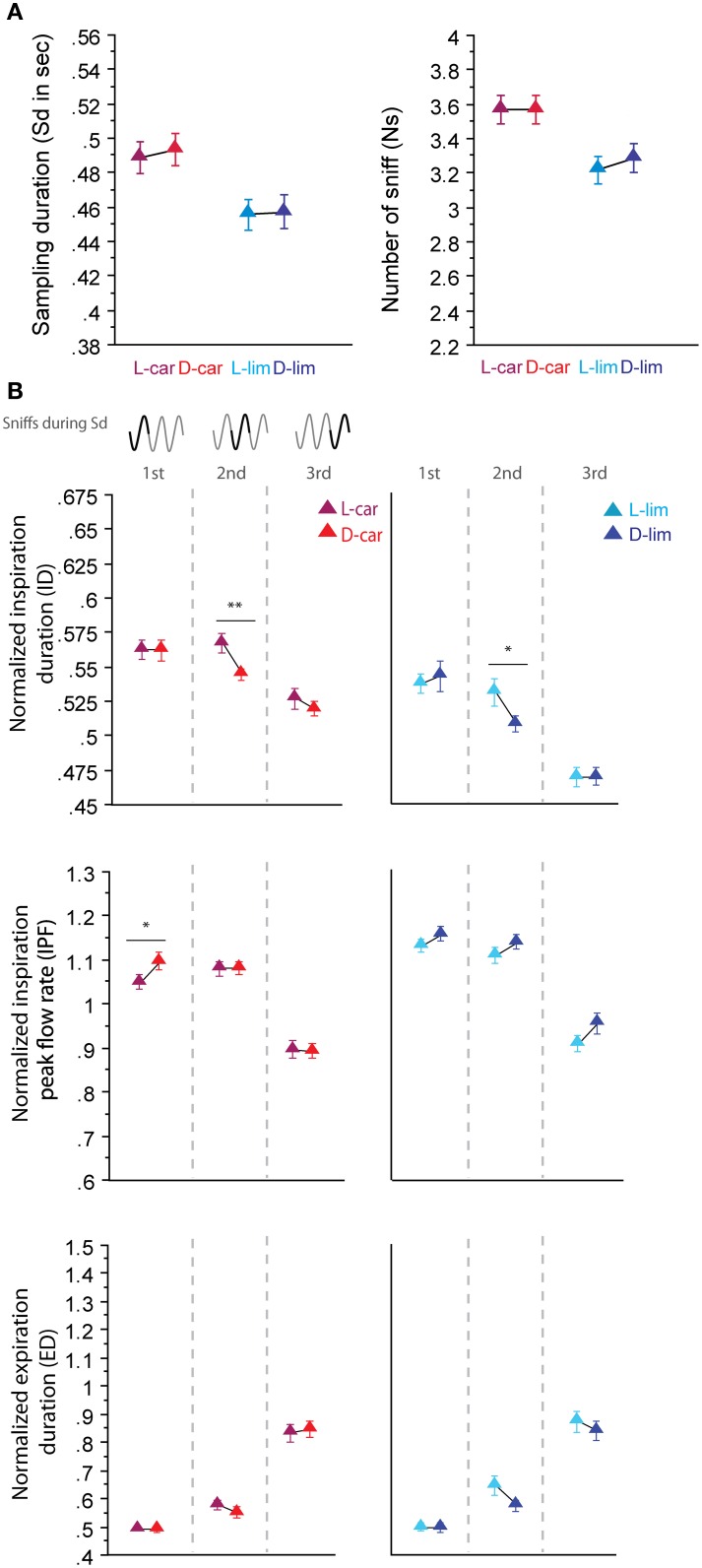
**Enantiomers are sniffed similarly. (A)** Global sampling parameters (mean ± s.e.m.), Sd (left) and Ns (right), for each odorant in each enantiomer odor pair: L-car (magenta)/D-car (red) *n* = 195; L-lim (cyan)/D-lim (dark blue) *n* = 211. **(B)** Modulation of sniff parameters in the first, second, and third cycles for (left to right) L-car/D-car and L-lim/D-lim. From top to bottom: mean (± s.e.m.) normalized ID, IPF, and ED. Same colors as in **(A)**. The number of trials for each odorant and cycle in L-car/D-car pair is: *n*_cycle 1_ = 195, *n*_cycle 2_ = 188, and *n*_cycle 3_ = 175 and in L-lim/D-lim pair is: *n*_cycle 1_ = 211, *n*_cycle 2_ = 201, and *n*_cycle 3_ = 162. Data were analyzed using a paired *t*-test; ^*^p < 0.05; and ^**^p < 0.01.

For the two pairs of enantiomers we tested (LS/LS and HS/HS pairs), the global sampling parameters were similar; animals sniffed these enantiomers with a similar Sd and a similar Ns [Figure 3A, Sd: L-car/D-car pair *t*_(194)_ = −0.323, *p* = 0.74; L-lim/D-lim pair *t*_(210)_ = −0.085, *p* = 0.93, Ns: L-car/D-car pair *t*_(194)_ < 0.001, *p* > 0.05; L-lim/D-lim pair *t*_(210)_ = −0.592, *p* = 0.56]. Similarly, an analysis of the fine sniffing parameters revealed few or no significant differences between enantiomeric odorants, regardless of the sorption properties, as shown in Figure 3B. For the L-car/D-car pair (Figure 3B, left), a significant difference appeared only in the ID during the second cycle [*t*_(187)_ = 2.994, *p* < 0.01] and in the IPF during the first cycle [*t*_(194)_ = −2.044, *p* < 0.05]. We also observed few differences between L-lim and D-lim (Figure 3B, right) with a significant difference only in the ID during the second cycle [*t*_(200)_ = 2.207, *p* < 0.05]. Thus, very similar molecules, such as the two pairs of enantiomers tested, induce similar sniffing strategies.

#### Pairs of non-enantiomeric odorants

**Figure 4:**
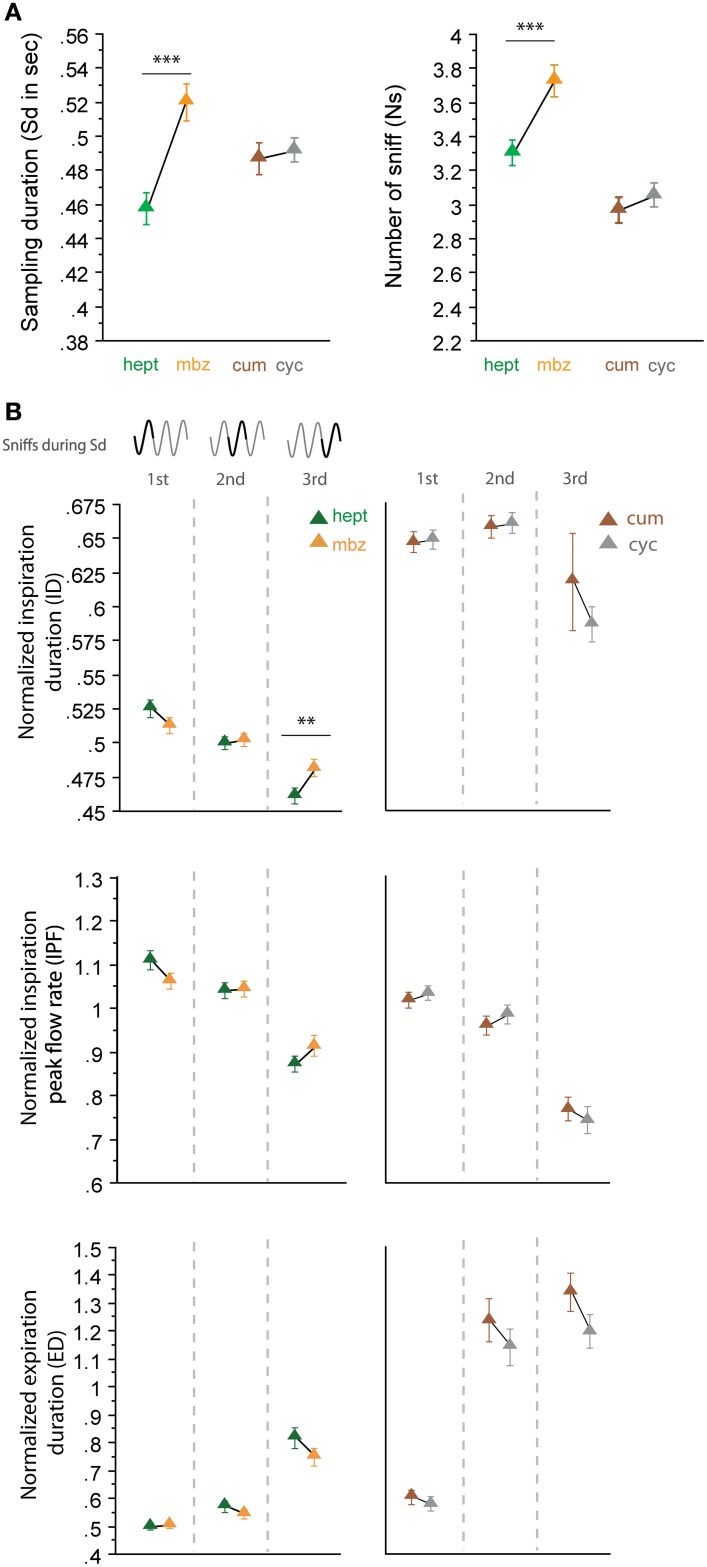
**Odorants with similar sorption properties but which are not enantiomers can induce different sniffing strategies. (A)** Global sampling parameters (mean ± s.e.m.), Sd (left), and Ns (right), for non-enantiomer odor pairs: hept (green)/mbz (orange) *n* = 168; cum (brown)/cyc (gray) *n* = 166. **(B)** Modulation of respiratory parameters in the first, second, and third cycles for (left to right) hept/mbz and cum/cyc. From top to bottom: mean (± s.e.m.) normalized ID, IPF, and ED. Same colors as in **(A)**. The number of trials for each odorant and cycle in hept/mbz pair is: *n*_cycle 1_ = 168, *n*_cycle 2_ = 163, and *n*_cycle 3_ = 144 and in cum/cyc pair is: *n*_cycle 1_ = 166, *n*_cycle 2_ = 160, and *n*_cycle 3_ = 110. Data were analyzed using a paired *t*-test; ^**^p < 0.01; and ^***^p < 0.001.

**Figure 5:**
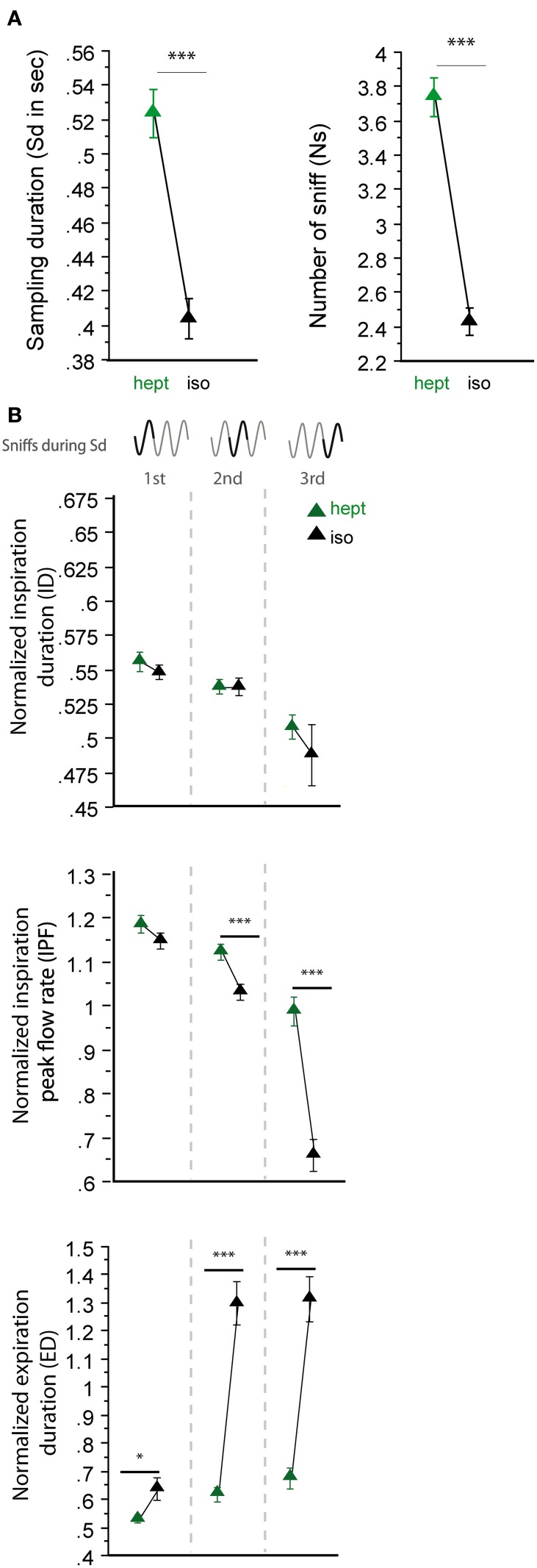
**Sorption is not the only parameter that determines global sniffing variations. (A)** Global sampling parameters (mean ± s.e.m.), Sd (left), and Ns (right), for non-enantiomer odor pair: hept (green)/iso (black) *n* = 150. **(B)** Modulation of respiratory parameters in the first, second, and third cycles for hept/iso. From top to bottom: mean (± s.e.m.) normalized ID, IPF, and ED. Same colors as in **(A)**. The number of trials for each odorant and cycle in hept/iso pair is: *n*_cycle 1_ = 150, *n*_cycle 2_ = 137, *n*_cycle 3_ = 65. Data were analyzed using a paired *t*-test; ^*^p < 0.05; and ^***^p < 0.001.

In a second step, we analyzed sniffing strategies when the animals had to discriminate between odorants that had similar sorption properties but were non-enantiomers (Figure 4). Here, we surprisingly observed more heterogeneous results; the odorants of the LS/LS pair (cum/cyc) were sampled similarly, but the odorants of the HS/HS pair (hept/mbz) were sampled differently. As shown in Figure 4, both global sampling parameters [Figure 4A, Sd: *t*_(165)_ = −0.513, *p* = 0.6084; Ns: *t*_(165)_ = −0.985, *p* = 0.3261] and fine parameters (Figure 4B, right) of individual sniffs were similar for cum and cyc. In contrast, both the Sd and Ns were significantly different between HS odorants hept and mbz [Figure 4A, Sd: *t*_(167)_ = −4.295, *p* < 0.001; Ns: *t*_(167)_ = −3.553, *p* < 0.001]. When we examined each cycle individually (Figure 4B, left), we observed only a few differences between hept and mbz with only one significant difference in the ID during the third cycle [*t*_(143)_ = −2.691, *p* < 0.01]. Thus, for the two different pairs of odorant molecules used that were non-enantiomers, we did not observe a sorption-based rule; molecules could be sniffed either similarly or differently even if they were endowed with similar sorption properties. This finding suggests that sorption is not the only parameter involved in sniffing adjustment and is confirmed by results from another pair of odorants, hept/iso, which have similar sorption properties (HS/HS, see Table 1) but differ in their vapor pressure. The results presented in Figure 5 reveal that both global sampling parameters [Figure 5A, Sd: *t*_(149)_ = 7.603, *p* < 0.001; Ns: *t*_(149)_ = 10.049, *p* < 0.001] and fine parameters of individual sniffs [Figure 5B, IPF second cycle: *t*_(136)_ = 3.551, *p* < 0.001; third cycle: *t*_(64)_ = 6.962, *p* < 0.001; ED first cycle: *t*_(149)_ = −2.553, *p* < 0.05; second cycle: *t*_(136)_ = −7.941, *p* < 0.001; third cycle: *t*_(64)_ = −7.952, *p* < 0.001] were significantly different between hept and iso. Thus, vapor pressure seems to enhance differences between sniffing strategies.

Taken together, these results show that odorants with similar sorption properties can be sniffed similarly (three pairs out of five) or differently (two pairs out of five). This finding suggests that sorption is not the only parameter involved in sniffing adjustment. Sniffing may not be restricted to analytical processing but instead may be adjusted synthetically, taking into account the pair in which the odorant is presented. We tested this possibility by analyzing sniffing strategies when the same molecule was presented in two different odor pairs.

### The same odorant induces different sniffing strategies when presented in different odor pairs: a synthetic strategy is likely

**Figure 6:**
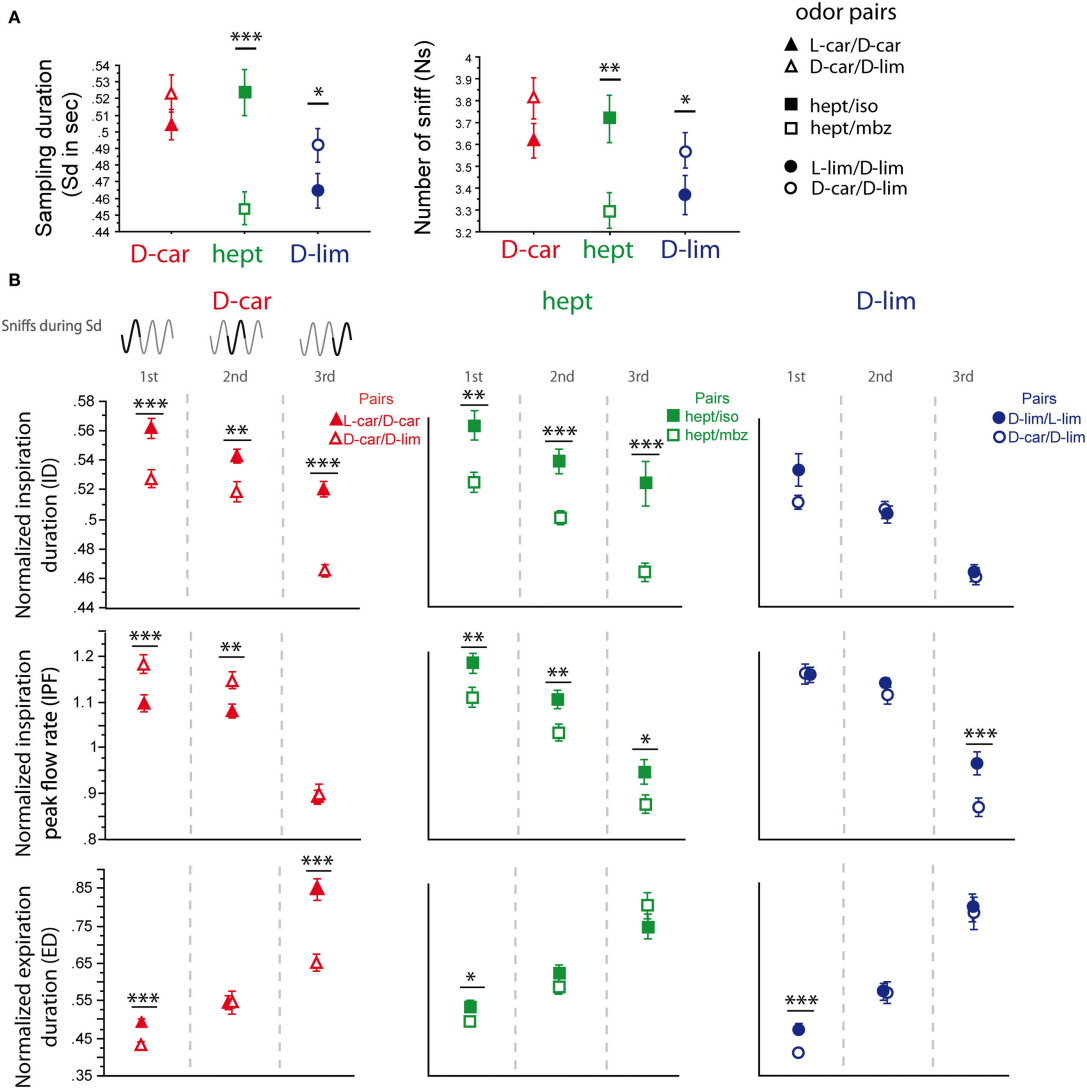
**Sniffing variations depend on the pair in which an odorant is presented. (A)** Global sampling parameters (mean ± s.e.m.), Sd (left), and Ns (right), for each odorant depending on the pair in which it was presented. D-car (red, *n* = 206) in the D-car/L-car pair (filled triangle) and in the D-car/D-lim pair (empty triangle); hept (green, *n* = 155) in the hept/iso pair (filled square) and the hept/mbz (empty square); D-lim (dark blue, *n* = 206) in the D-lim/L-lim pair (filled circle) and the D-car/D-lim pair (empty circle). **(B)** Modulation of respiratory parameters in the first, second, and third cycles for the same odorant presented in two different pairs. Left: D-car in the enantiomer pair or the D-car/D-lim pair; middle: hept in the hept/iso pair or the hept/mbz pair; right: D-lim in the enantiomer pair or the D-car/D-lim pair. From top to bottom: mean (±s.e.m.) of the normalized ID, IPF, and ED. The number of trials for each odorant and cycle is: D-car: *n*_cycle 1_ = 206, *n*_cycle 2_ = 197, and *n*_cycle 3_ = 181; hept: *n*_cycle 1_ = 155, *n*_cycle 2_ = 148, and *n*_cycle 3_ = 121; D-lim: *n*_cycle 1_ = 206, *n*_cycle 2_ = 200, and *n*_cycle 3_ = 157. Data were analyzed using a paired *t*-test; ^*^p < 0.05; ^**^p < 0.01; ^***^p < 0.001.

Three odorants, D-car, hept and D-lim, were presented in two different pairs (Figure 6), which allowed us to compare the sniffing pattern for the same odorant when it was presented in two different pairing contexts (see Materials and Methods). As shown in Figure 6A, except for D-car [Sd: *t*_(205)_ = −1.424, *p* = 0.156; Ns: *t*_(205)_ = −1.618, *p* = 0.107], the global sampling parameters were significantly affected by the pair in which the odorant was presented. Animals took more sniffs and remained longer in the odor port when hept was presented in the hept/iso pair than in the hept/mbz pair [Figure 6A, Sd: *t*_(154)_ = 4.335, *p* < 0.001; Ns: *t*_(154)_ = 3.104, *p* < 0.01]. Similarly, animals took more sniffs and remained longer in the odor port when D-lim was presented in the D-car/D-lim pair than in the L-lim/D-lim pair [Sd: *t*_(205)_ = −2.191, *p* < 0.05; Ns: *t*_(205)_ = −1.976, *p* < 0.05].

We further measured the respiratory cycle parameters. Here too, we observed significant variations in sniffing cycle parameters depending on the pair in which the odorant was presented. As shown in Figure 6B, D-car induced significant differences in the ID, IPF, and ED parameters when presented in different pairs. Animals sniffed longer (higher ID and ED) and with a lower IPF when D-car was presented in the L-car/D-car pair than in the D-car/D-lim pair [Figure 6B, left; ID: first cycle *t*_(205)_ = 3.734, *p* < 0.001; second cycle *t*_(196)_ = 3.164, *p* < 0.01; third cycle *t*_(180)_ = 8.022, *p* < 0.001; IPF: first cycle *t*_(205)_ = −3.440, *p* < 0.001; second cycle *t*_(196)_ = −3.165, *p* < 0.01; ED: first cycle *t*_(205)_ = 4.702, *p* < 0.001; third cycle *t*_(180)_ = 5.322, *p* < 0.001]. Similarly, hept induced a significant difference in the ID and IPF of the three cycles and in the ED of the first cycle when presented in two different pairs [Figure 6B, middle; ID: first cycle *t*_(154)_ = 3.132, *p* < 0.01; second cycle *t*_(147)_ = 3.775, *p* < 0.001; third cycle *t*_(120)_ = 3.641, *p* < 0.001; IPF: first cycle *t*_(154)_ = 2.77, *p* < 0.01; second cycle *t*_(147)_ = 2.687, *p* < 0.01; third cycle *t*_(120)_ = 2.27, *p* < 0.05; ED: first cycle *t*_(154)_ = 2.041, *p* < 0.05]. In this case, animals sniffed hept with a higher ID, ED, and IPF in the hept/iso pair than in the hept/mbz pair. For D-lim, the respiratory cycle variations were more modest, with significant differences only in the IPF of the third sniff [Figure 6B, right; *t*_(156)_ = 3.836, *p* < 0.001] and in the ED of the first cycle [*t*_(205)_ = 3.891, *p* < 0.001].

As a whole, these results show that the same odorant presented in two different odor pairs was sniffed differently by changing the global sampling and/or the individual sniff cycle features. This finding suggests that there is no absolute sniff pattern corresponding to one odorant or a category of odorants but rather a relative pattern based on the pairing context of the odorants.

## Discussion

By finely measuring the sniff parameters of rats performing a two-alternative odor choice discrimination task, we showed that molecules with similar sorption properties can be sniffed similarly (three out of five odor pairs tested) or differently (two out of five odor pairs tested) indicating that sniffing variations are not only governed by odorant sorption. Further, we provided new evidence that sniffing adjustment is a synthetic process dependent on the pair of odorants presented in the discrimination task.

### Relation between sniffing pattern and sorption properties

Based on pioneering studies of OE function, the zonation hypothesis was proposed to explain the role of sniffing in olfaction (Schoenfeld and Cleland, 2005, 2006). This hypothesis proposes that sniffing optimizes the deposition of odorant molecules through the OE and is dependent on the sorption properties of the odorant molecules. Rojas-Líbano and Kay (2012) showed that odorants with different sorption properties induce different sniffing strategies. However, they did not show that odorants with similar sorption properties induce similar sniffing patterns. For the first time, we showed that enantiomers were sniffed similarly, at least true for the two odor pairs used (Figure 3), confirming the relationship between molecular properties and sniff parameters. However, we also demonstrated that such a strong relationship does not exist for non-enantiomeric odorants with similar sorption properties; some non-enantiomers were sniffed similarly, whereas others were sniffed differently (Figure 4). This observation may explain why significant sniffing variations between odorants differing in their sorption properties were not observed by Cenier et al. (2013). Several factors can account for the lack of a strict relationship between sorption properties and sniffing strategy in non-enantiomeric molecules. First, as shown recently by Scott et al. (2014), the electroolfactogram responses of medial and lateral recesses of the OE—which are anatomically optimized for odorants with different solubility—are differently affected by high nasal flow rates in active sniffing. Thus, using a larger set of odorants and/or odorants specifically activating the central zone may have led to different results. Second, physicochemical properties of the odorant other than sorption are likely components of sniff adjustment. For example, we showed that a difference in vapor pressure seems to enhance the difference of sniffing strategy between two odorants with similar sorption properties (Figure 5). Third, other factors related to the interaction between odorant molecules and their receptors may be involved, such as molecule/receptor affinity, molecule/odorant binding protein interactions (Pelosi, 2001), the number of receptors accessible to the molecule or enzymatic degradation of the odorant molecule (Thiebaud et al., 2013). Fourth, it is highly likely that sniffing is not adjusted in an absolute manner that is dependent on the properties of an individual odorant but instead takes into account the context (here, the pair of odorants) in which an odorant is presented.

### Sniffing as a synthetic strategy

We observed that a same odorant could induce different sniffing pattern depending on the odor pair in which it is presented. It seems that, in our experimental conditions, sniff modulation is a synthetic process taking into account not only the individual odorant properties but also the context in which an odorant is presented. This result was unexpected, and the physiological role of such a strategy is therefore of interest. We propose that a synthetic strategy may optimize sniffing to achieve the maximal decorrelation of OE activation between odorants. Indeed, in our task, the goal of the animal was to act rapidly and successfully to gain a reward. In such conditions, the aim is not to clearly identify the odorant but rather to find the most reliable clue to make the correct decision. Thus, the animal may adjust its sniffing to achieve the greatest difference between the two OE activation patterns and could thus adopt a type of intermediate sniffing pattern. This hypothesis could explain why the same odorant was sniffed differently when presented in two different pairs. We do not claim that the olfactory system does not use the sorption properties of the odorants. Rather, the system likely uses these properties in combination with other contextual information to quickly perform correct odorant discrimination.

Our observation that sniffing is synthetically modulated fits well with the concept that sniffing is modulated by higher functions such as emotion (Hegoburu et al., 2011), context (Wesson et al., 2008a), social behavior (Wesson, 2013), or attentional demand (Plailly et al., 2008). This concept implies that we could observe different results using other experimental conditions such as a different behavioral paradigm. Interestingly, the activity of the first olfactory brain relay, the olfactory bulb, is modulated by context and learning (Kay and Laurent, 1999; Doucette et al., 2011). The dependence of sniff modulation and olfactory bulb activity on contextual clues likely originates in the complex relationship among perception, motor, motivation and respiratory pathways (Clarke and Trowill, 1971; Ikemoto and Panksepp, 1994; Kepecs et al., 2007). For example, inputs from these centers may modify olfactory bulb activity as a function of attention, motivation and learning (Gray and Skinner, 1988). These centers may also act on the respiratory system and be part of the network involved in controlling olfactomotor action.

### Sniffing adjustment: a rapid and fine olfactomotor act

The olfactomotor act could be defined as a modulation of sniffing by the olfactory system. Different authors have shown that odorant presentation can modify or trigger sniffing (Welker, 1964; Alberts and May, 1980) as well as induces a concomitant modification of the firing pattern of respiratory center neurons (du Pont, 1987). The effect on the sniff is extremely fast and appears in the 50 ms following the olfactory bulb activation by an odorant (Wesson et al., 2008a,b) or even earlier if the olfactory bulb is electrically stimulated (45 ms, Monod et al., 1989). In humans, Johnson et al. (2003) also showed that sniffing could be quickly adapted, i.e., within 160 ms, depending on odorant concentration. In accordance with these observations, we showed that sniffing can vary during the first cycle following odor onset (Figures 3–5). The animal thus has the possibility to adjust his sniffing very quickly. Moreover, when we analyzed the global sniffing parameters (Sd and Ns) and the fine parameters of the first, second, and third cycles (ID, IPF, and ED), we observed that all these parameters can be modulated individually or concomitantly. As a comparison, sampling frequency and flow rate can act either independently or synergistically on bulbar output to shape the neuronal message (Courtiol et al., 2011; Esclassan et al., 2012). The animal could use each parameter independently or combine some of them to achieve specific functions. Those various modulations reveal a high flexibility in sniff adjustment and the olfactory system may need to use all the possible adjustments to improve odor representation in the olfactory center and therefore odor discrimination.

## Conclusions

In conclusion, we showed that rats use a synthetic sniffing strategy that considers the pair of odorants to be discriminated. The system likely uses the odorant properties in combination with other contextual information to quickly perform correct odorant discrimination. Our results provide an additional argument demonstrating that sniffing is a specific, quickly and finely adapted sensorimotor act. Future studies will investigate the extent to which sniffing variations help or are mandatory to perform correct olfactory discrimination.

### Conflict of interest statement

The authors declare that the research was conducted in the absence of any commercial or financial relationships that could be construed as a potential conflict of interest.
